# Hepatoblastoma: current understanding, recent advances, and controversies

**DOI:** 10.12688/f1000research.12239.1

**Published:** 2018-01-15

**Authors:** Piotr Czauderna, Hanna Garnier

**Affiliations:** 1 Department of Surgery and Urology for Children and Adolescents, Medical University of Gdansk, Ul. Nowe Ogrody 1-6, 80-803 Gdansk, Poland

**Keywords:** hepatoblastoma, liver transplantation, chemotherapy, PRETEXT, surgical treatment, biology

## Abstract

**Introduction**: Hepatoblastoma (HB) is the most common primary malignant liver neoplasm in children. Its increasing survival rate is related to the progress in modern imaging, surgical techniques, and new chemotherapy regimens.

**Clinical approach**: One of the past achievements was the development of the pretreatment extension of disease (PRETEXT) system. Gradually, the HB therapeutic approach has become more individualized with better stratification of patients.

**Controversies**: These include the need for preoperative chemotherapy and its optimal duration; intensity of preoperative chemotherapy required for locally advanced cases (PRETEXT 4); optimal surgical treatment for locally advanced tumors: aggressive hepatic resections versus liver transplantation; the role of postoperative chemotherapy in the post-transplant setting; the timing and role of metastasectomy in patients with disseminated disease who undergo partial liver resection; and the prognostic significance of several HB pathology variants.

**Hepatoblastoma biology**: Beta-catenin mutations and the beta-catenin/Wnt pathway play an important role in HB development. There have been at least two molecular signatures in HB published. Unluckily, all of these findings are based on relatively small clinical series and require confirmation.

**Conclusion**: The treatment of HB started from one and the same therapy for all patients and aimed at increased treatment individualization, but the future seems to lie in biology-driven patient-tailored therapies.

## Introduction

Hepatoblastoma (HB) is the most common primary malignant liver neoplasm in children ^1^. In the vast majority of cases, it is associated with elevated alpha-fetoprotein (AFP), which is helpful in diagnosis and monitoring response to treatment as well as the follow-up. In the modern era, HB is associated with very good survival, in the range of 70–80%, although before the introduction of chemotherapy (CHT) it was below 30% ^2^. This is related to the progress in modern imaging, surgical techniques including liver transplantation (LTX), and efficient CHT regimens ^3, 4^.

## Clinical approach

It seems that CHT progress was associated with its intensification and the introduction of the more time-frequent use of cisplatin, which supposedly is the key drug in HB ^5^. However, not all patients do equally well. Metastatic cases, especially HBs associated with low AFP levels (<100 ng/mL), are associated with decreased survival. Particularly the latter subgroup has a truly dismal outcome ^6^. Furthermore, doxorubicin is a very important drug in HB treatment, especially in advanced-stage patients. The Children’s Oncology Group (COG) found that a platinum-alone regimen for stage III and IV patients was associated with significantly increased failure rate and therefore was substituted with a regimen including doxorubicin which brought very good outcomes, especially in stage III patients. However, it is possible that this observation resulted from the fact that in the quoted regimen cisplatin was de facto deintensified because of its partial substitution with carboplatin, which seems to be less active. Nevertheless, the International Childhood Liver Tumours Study Group (SIOPEL group) also includes doxorubicin as an integral part of treatment for patients at very high risk ^7^.

Several issues, especially of prognostic significance, have recently been clarified by the efforts of the Children’s Hepatic tumors International Cooperation (CHIC) group ^1^. The CHIC group has developed an international clinical database collecting 1,605 HB cases treated in prospective multicenter trials under the auspices of the American COG, the Japanese Pediatric Liver Tumours study group (JPLT), and the European SIOPEL group. Several key factors, which were considered doubtful (that is, tumor rupture at diagnosis, vascular invasion, and multifocality), have been proven to affect patient survival. Although the highest incidence of HB occurs in children younger than 5 years old, HB cases in older children have been published. The influence of older age at diagnosis on treatment outcome and tumor histology in HB has not been well studied. However, it seems from the CHIC effort that older HB patients do much worse, but the reason for this is not entirely clear. It is possible that this is associated with different histology/biology of the tumor as well as its different response to standard therapy ^8, 9^.

Another achievement of the SIOPEL group was the development of the PRETEXT system, which served to assess pretreatment extension of disease within the liver. Subsequently, it became an accepted worldwide standard ^10, 11^.

Gradually, the HB therapeutic approach has become more individualized with better patient stratification based upon initial clinical features. Although initially all HBs were treated in the same way, later at least two new patient categories emerged:

1. Standard-risk HB, which is entirely limited to the liver but leaving at least one of its four sections free (that is, involving no more than three out of four of its sections, thus being potentially resectable) and associated with elevated AFP levels (>100 ng/mL).

2. High-risk HB, which is metastatic or involving the whole liver (PRETEXT 4 tumors) or has extrahepatic/vascular extension or is associated with low AFP or a combination of these factors.

## Controversies

Nevertheless, several areas of controversy persist. This applies mainly to the following:

1. The need for preoperative CHT and its optimal duration. Owing to differences between the European approach, which tends to rely on preoperative CHT in every case, and the American one, which favors primary tumor resection, it is unclear what the best indications for preoperative CHT are and what the optimal number of courses is. Both approaches resulted in similar outcome. The recently proven improvement in survival of metastatic cases based upon SIOPEL 4 intensified cisplatin approach ^5^. However, it seems that, in most cases, standard cisplatin monotherapy is sufficient, and this was convincingly proven by the SIOPEL 3 study ^12^. This controversy may be resolved by a new Pediatric Hepatic Malignancy International Therapeutic Trial (PHITT), which is organized jointly by COG, JPLT, and SIOPEL, combining experiences and past approaches. In this study, two versus four courses of preoperative CHT will be compared in standard-risk HB.

2. Intensity of preoperative CHT required for locally advanced cases (PRETEXT 4). It is unclear whether, in PRETEXT 4 tumors, more intense preoperative CHT makes sense since many of them will require LTX anyhow and hence toxic effects of aggressive CHT might be better avoided. On the other hand, it is known from the previous studies that about 30–50% of such tumors can be downstaged because of CHT and eventually undergo partial hepatectomy instead of LTX ^11^.

3. Optimal surgical treatment for locally advanced tumors: aggressive hepatic resections versus LTX. It is clear from the basic study by Otte *et al*. that salvage LTX in HB (for example, performed for local relapse or incomplete previous tumor resection) is associated with significantly inferior survival when compared with primary LTX (70% versus less than 30%) ^12^. This observation became a cornerstone to favor LTX over aggressive liver resections. However, one must remember that despite good short- and medium-term results, LTX is not free from its own co-morbidities such as secondary neoplasms and the lifelong (at least in the majority of cases) need for immunosuppression with all its potential negative side effects and consequences. Additionally, recently published SIOPEL observations have shown that microscopic residuum in HB is not associated with decreased patient survival ^13^. Again, the PHITT may help to answer this question in the future, although it may be difficult because of the partially subjective and surgeon-dependent nature of the tumor’s resectability assessment.

4. The role of postoperative CHT in the post-transplant setting. At the moment, there is no clear policy and evidence base regarding this aspect. Several studies documented equally good results regardless of whether subsequent CHT was used after transplant ^14– 16^. There is certainly a fear that CHT may contribute to an increased risk of LTX complications. Nevertheless, it seems that nowadays most transplant centers tend to favor the use of postoperative CHT.

5. The timing and role of metastasectomy in patients with disseminated disease who undergo partial liver resection. Modern CHT is very successful in the eradication of lung metastases in HB patients; a 90% response rate was observed in the recent SIOPEL 4 study. Traditionally, in the LTX setting, pulmonary metastases, which persisted after induction CHT, had to be resected before LTX ^4^. However, in patients who underwent partial liver resection, a different policy was used: pulmonary metastasectomy followed hepatectomy and usually was preceded by one or two CHT courses. This approach resulted from two facts: a fear that hepatic growth factors are excreted in the regeneration process after hepatectomy which may contribute to the development/growth of lung metastases and a chance for complete regression of metastases in the course of postoperative CHT ^17^. This issue has never been properly studied and remains unresolved.

6. There are also some remaining controversies over whether CHT alone is sufficient for the clearance of pulmonary metastases in patients undergoing LTX. Some surgeons still require surgical exploration of those patients to prove that they have no viable HB deposits in their lungs before undergoing a transplant.

7. Treatment for the refractory or relapsed patients is also far from being standardized, and several regimens, including irinotecan alone, vincristine/irinotecan, gemcitabine/oxaliplatin, and docetaxel monotherapy, have been tested. This is somewhat dependent on the first line of therapy. If a patient was treated with cisplatin alone, then carboplatin/doxorubicin as the second line may be a very successful and reasonable option.

8. Prognostic significance of several HB pathology variants. In the past, several HB variants have been postulated to be associated with inferior survival of patients (that is, microtrabecular or anaplastic variant) ^18, 19^. On the contrary, pure fetal well-differentiated HB (PF-HB) was associated with very good survival which was proven in subsequent COG studies. In the North American trials, there have been no recurrences observed in PF-HB patients treated with surgery alone ^20, 21^. Some researchers postulated prognostic significance of percentage of tumor necrosis in the resected tumor, which can be predictive for the outcome; however, these findings have never been confirmed in larger series. According to Venkatramani *et al*., patients who had at least 30% necrosis in the resected tumor had a better event-free survival compared with the group having less than 30% necrosis ^22^. Owing to previous differences in pathology HB classifications between various international study groups, the issue of prognostic pathology subtype significance was very difficult to study, even using the CHIC approach. However, after the development and publication of a new international classification of pediatric liver tumors, that effort is underway ^23^. Currently, all CHIC database patients for whom pathology material was available and could be scanned and digitalized are being re-reviewed by the international CHIC pathology committee. This analysis should be finished by the end of the year.

## Hepatoblastoma biology

Owing to huge progress in basic and translation research, our understanding of HB biology has improved greatly. There has been a continual search for possible molecular prognostic factors that may help in the diagnosis and treatment of this neoplasm. However, correlation between biology and pathology remains unclear. Two main histological types of HB occur: epithelial and mixed epithelial/mesenchymal. The epithelial type is further divided into subtypes, such as fetal, embryonal, combined fetal and embryonal or macrotrabecular and small cell type. The mixed type is characterized by the presence of some extra-mesenchymal elements, such as cartilage or osteoid ^24, 25^. Histological differences between various subgroups of HB, as well as the fact that 40% of tumor samples contain both epithelial and mesenchymal elements, may be explained by the cellular basis of this neoplasm. HB arises not only from primary hepatoblasts but also from less differentiated cells. Hepatic stem cells and human fetal liver multipotent progenitor cells (hFLMPCs), which are poorly differentiated, are able to convert into a variety of tissues, such as hepatocytes, bone, fat, or bile ducts. Thus, a suspected origin of HB from hFLMPCs may explain its variety ^26^.

It is widely known that beta-catenin plays a crucial role in the development of various human organs. According to the newest research, it seems that beta-catenin mutations and the beta-catenin/Wnt pathway play an important role in HB development. According to research by Tan *et al*. in a murine model, where the CTNNB1 was knocked out, partial hepatectomy leads to hepatocyte proliferation and hepatic cell growth ^27^. Activation of beta-catenin, in a physiological context, is mainly regulated by the Wnt pathway ^28^. Its abnormalities collectively account for most of the genetic defects in HB. In addition, the accumulation of beta-catenin is observed in almost all HB cases. The new cooperative role of beta-catenin signaling and Yap signaling in HB pathogenesis should be included in future studies as well. Tao *et al*., in their research in mice, showed that overexpression of activated forms of the above proteins leads to rapid tumor development. Moreover, it seems that the results of their study may identify new potential therapeutic targets ^29^.

There have also been at least two molecular signatures in HB published ^30, 31^. Unluckily, all of these findings are based on relatively small clinical series and require confirmation, which again may come through the biological part of the PHITT.

## Conclusions

The story of the treatment of HB has been a fascinating journey starting from one and the same therapy for all patients and aiming at increased treatment individualization, but the future seems to lie in biology-driven patient-tailored therapies.
